# Chill and Fever

**Published:** 1871-02

**Authors:** 


### CHILL AND FEVER

Is the all-pervading blight within fifty miles of New York City, causing multitudes who go to the country for health to return in the fall of the year saturated with disease, which the slightest indiscretion or exposure develops into some troublesome fever or mysterious form of sickness. Chill and fever, intermittents, and the old-fashioned name of

### FEVER AND AGUE, are one and the same disease, arising from the one cause of low, damp lands or stagnant water, whether in the shape of mill-ponds, lakes, sluggish streams, or pools of water here and there in the beds of half-dried creeks and rivulets. Many neighborhoods are the hot-beds of disease in summer, because the ponds are not filled up or the lowlands drained. If standing water is drained in warm weather, it exaggerates the sickness a thousand fold, because the slimy bottom is exposed to the hot sun, and the deadly miasm is generated in incredible quantities and of concentrated virulence.

The time for draining ponds or other forms of stagnant water is midwinter. Then fill up or plough and cultivate in early spring, keeping all the drains free. To show what intelligence and public spirit will do through a single individual, it may be stated that a gentleman living near New York, observing that there was a great deal of sickness near his country place every autumn, quietly purchased all the ponds in his neighborhood, to the great astonishment and wonderment of everybody. He subsequently drained them, and, when the bottoms were dry, ploughed them deeply, thoroughly, and well; then sowed his seed. The fall gave a crop which surprised "everybody;" such a yield had never been heard of in all that section of country; and last September, when Staten Island, New Haven, Poughkeepsie, and

### ALL THE REGION ROUND ABOUT

New York City, were overrun in many parts with chill and fever to an unremembered extent, the village of West Farms is not known to have had a single case. In many parts of our country intelligent enterprise can prevent disease, produce unheard-of crops, and add one hundred per cent. to the value of adjoining lands.

